# Structure-based drug design studies of UDP-*N*-acetylglucosamine pyrophosphosrylase, a key enzyme for the control of witches’ broom disease

**DOI:** 10.1186/1752-153X-7-48

**Published:** 2013-03-05

**Authors:** Manoelito C Santos Junior, Sandra Aparecida de Assis, Aristóteles Góes-Neto, Ângelo Amâncio Duarte, Ricardo José Alves, Moacyr Comar Junior, Alex Gutterres Taranto

**Affiliations:** 1 Departamentos de Saúde, Feira de Santana, Feira de Santana-BA 44031-460, Brazil; 2 Ciências Biológicas, Feira de Santana, Feira de Santana-BA 44031-460, Brazil; 3 Tecnologia, Programa de Pós-Graduação em Biotecnologia, Universidade Estadual de Feira de Santana, Feira de Santana-BA 44031-460, Brazil; 4 Universidade Federal de Minas Gerais, Faculdade de Farmácia, Departamento de Produtos Farmacêuticos, Av. Antônio Carlos, 6627, Pampulha 31270-901, Belo Horizonte, MG, Brazil; 5 Laboratório de Modelagem Molecular, Programas de Pós-Graduação em Biotecnologia e Ciências Farmacêuticas, Campus Centro Oeste (CCO), Universidade Federal de São João Del-Rei, Divinópolis-MG 35501-296, Brazil

**Keywords:** Docking, Molecular dynamics, Pyrophosphorylase, *Moniliophthora perniciosa*

## Abstract

**Background:**

The witches’ broom disease is a plague caused by *Moniliophthora perniciosa* in the *Theobroma cacao,* which has been reducing the cocoa production since 1989. This issue motivated a genome project that has showing several new molecular targets, which can be developed inhibitors in order to control the plague. Among the molecular targets obtained, the UDP-*N*-acetylglucosamine pyrophosphorylase (UNAcP) is a key enzyme to construct the fungal cell wall. The inhibition of this enzyme results in the fungal cell death.

**Results:**

The results show that the molecular recognition of the enzyme with the substrates occurs mainly by hydrogen bonds between ligands and Arg116, Arg383, Gly381, and Lys408 amino acids; and few hydrophobic interactions with Tyr382 and Lys123 residues.

**Conclusions:**

Among the compounds analyzed, the NAG5 showed the best binding energy (−95.2 kcal/mol). The next steps for the control of witches’ broom plague involve the synthesis and biological evaluation of these compounds, which are in progress.

## Background

In 1989, the economy of Bahia State in Brazil was strongly impacted and cocoa production dropped drastically because of the emergence and proliferation of the fungus *Moniliophthora perniciosa*, a plague known as witches’ broom [1]. Consequently, Brazil began to import cocoa [2,3]. Several efforts have been made to contain the spread of witches’ broom in Bahia. However, most of them have failed for several reasons, particularly the lack of genetic resistance during the initial contact with this plague [4]. The progress of the pest has been slowed using agricultural practices such as phytosanitary pruning, fertilisation and application of copper fungicides. However, the cost of fungicide application has made these practices impossible for most farmers [4,5]. These data motivated the search for an alternative chemical control for witches’ broom.

Several chemical compounds have been tested for their capacity to prevent or eradicate witches’ broom. The results, however, have not been satisfactory [6]. Inhibitors of bacterial cell wall biosynthesis, such as penicillin and cephalosporin, have shown good results in the control of bacterial infections. Similarly, the cell walls of fungi are promising targets for the development of potent antifungals [7-12]. The main component of the fungal cell wall is chitin, which is formed from a key precursor denoted by UDP-*N*-acetylglucosamine. This precursor is formed by a reaction between *N*-acetylglucosamine-1-phosphate and uridine*-*5^′^-triphosphate (UTP), which is catalyzed by UDP-*N*-acetylglucosamine pyrophosphorylase (UNAcP). The inhibition of UDP-*N*-acetylglucosamine formation affects the synthesis of chitin. Therefore, fungal cell wall synthesis cannot take place, resulting in fungal cell death [13-15].

The conformation of UTP in the active site of UNAcP is similar in all enzymes deposited in the Protein Data Bank (PDB) [16]. The UTP binds in the central region of the enzyme, making contact with a loop formed by residues from Asp221 to Leu226. The sugar moiety is stabilized by hydrogen bonds formed by Ala261 and Asp417 residues. The *N*-acetyl moiety establishes hydrogen bonds with the Asn223, Glu303 and His331 and a hydrophobic interaction with Phe381 and Phe383 [16]. However, this hydrophobic pocket is not present in other enzymes in the active site of pyrophosphorylases from *M. perniciosa*[14].

This work aimed to perform studies of molecular docking followed by molecular dynamics simulations of the enzyme UDP-*N*-acetylglucosamine pyrophosphorylase from the fungus *M. perniciosa*. The goal is the development of new inhibitors of the pyrophosphorylase enzyme that can be used for management of this pathogen.

## Results and discussion

Three points were determined to be important for molecular recognition by the enzyme substrate: the pentose, uracil and *N*-acetylglucosaminyl moieties. In addition, the enzyme has two substrates, UTP and *N*-acetylglucosamine-1-phosphate [14-17]. In analysing the molecular recognition, it can be observed that the pentose and nucleoside moieties establish hydrogen bonds with the residues of the second half of the central loop of the UNAcP (residues from Val68 to Val260). *N*-acetylglucosamine is also recognized by hydrogen bonds, and it is possible to observe a hydrophobic interaction in human UNAcP that occurs between two phenylalanines (381 and 383) [16].

**Figure 1:**
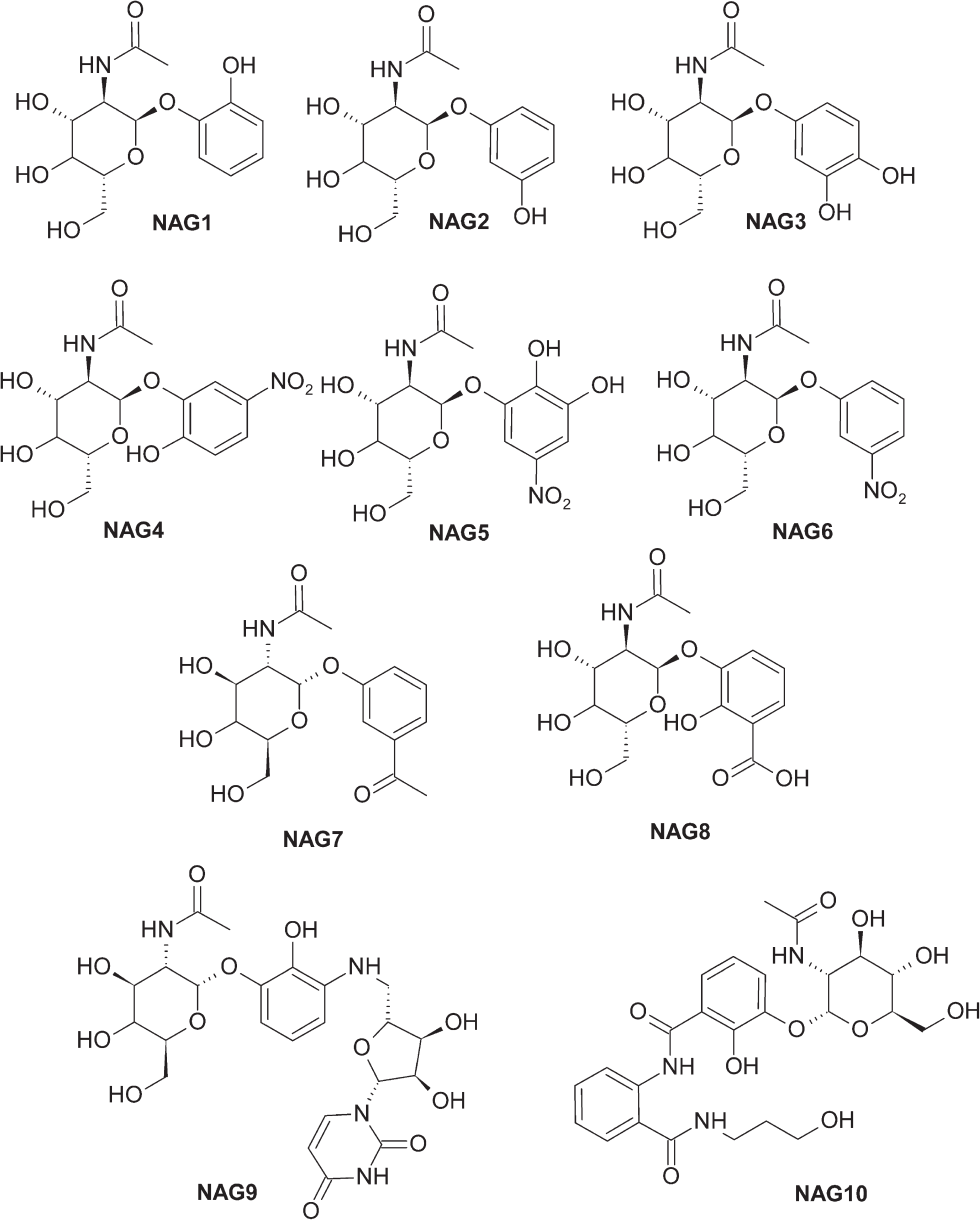
Structure of the ten potential inhibitors.

Information about the active site of the enzyme is essential for the development of ligands [18]. Based on this data regarding the compounds that are molecularly recognized by the enzyme, ten potential UNAcP inhibitors were proposed for the enzyme (Figure 1). The first step of the docking studies involves a detailed analysis of the binding site and a description of all aspects in relation to affinity and selectivity [18]. The active site in the previous model built of UNAcP from *M. perniciosa* is highly conserved compared with the crystallographic structure 1JV1 [16]. However, the regions between 379–384 and 403–409 residues showed no similarity, which is highlighted by the hydrophobic pocket formed by residues Phe381 and Phe383. They are absent in the constructed model [14].

**Figure 2:**
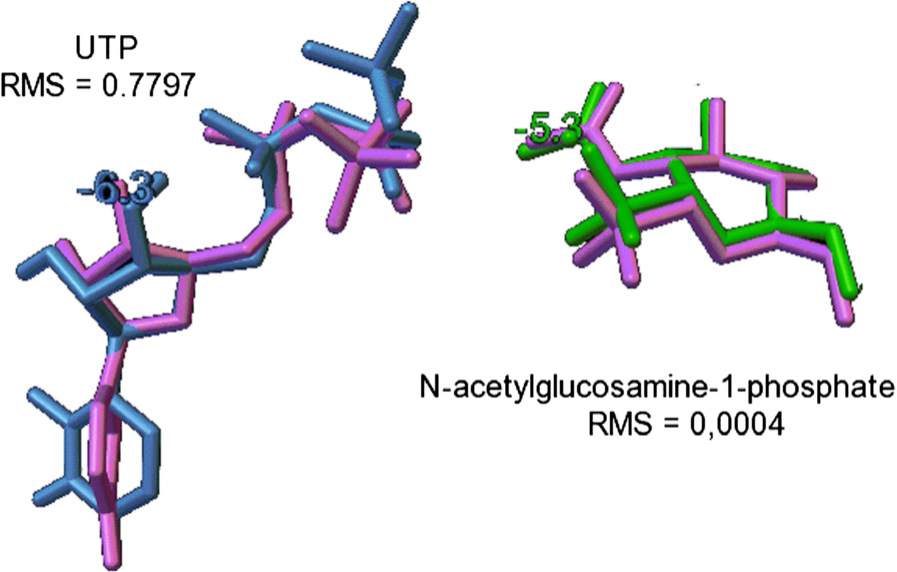
**Redock structures used as reference together with the value of the RMS and binding energy.** In pink: reference structures; blue: UTP and green: the N-acetylglucosamine-1phosphate. Image constructed in the program AutoDockTools 1.5.6rcz2. RMS calculated using MacPymol program.

The second step for docking simulation is the redock of crystallographic ligand to validate the process. The conformations of redock are shown in Figure 2. As can be seen, RMS values of 0.00 Å and 0.78 Å were found for *N*-acetylglucosamine-1-phosphate and UTP, respectively. RMS values below 2 Å are considered acceptable [19-21]. These values were satisfactory, indicating that the structures were well anchored. The present spatial positioning was very near the structures that were used as references, thereby indicating that the size and location of the grid box were satisfactory. The binding energy of redocking structures, *N*-acetylglucosamine-1-phosphate and UTP, was −5.3 and −6.3 kcal/mol, respectively. Table 1 shows the docking results of all compounds in the active site of UNAcP. The compound with the most affinity is NAG9 (−9.1 kcal/mol), whereas the compound with the least affinity is NAG5 (−7.0 kcal/mol). Next, the docking geometry was used for the MD simulations.

**Table 1:** Binding energy estimated by Autodock Vina and AMBER11

**Binding energy (Kcal/mol)**		
**Compounds**	**Autodock Vina**	**AMBER11**
NAG1	−7.2	−59.0
NAG2	−7.3	−88.2
NAG3	−7.8	−25.5
NAG4	−7.2	−52.1
NAG5	−7.0	−95,2
NAG6	−7.9	−8.0
NAG7	−8.0	−72.2
NAG8	−7.7	−17.6
NAG9	−9.1	−83.7
NAG10	−8.8	−22.4

**Figure 3:**
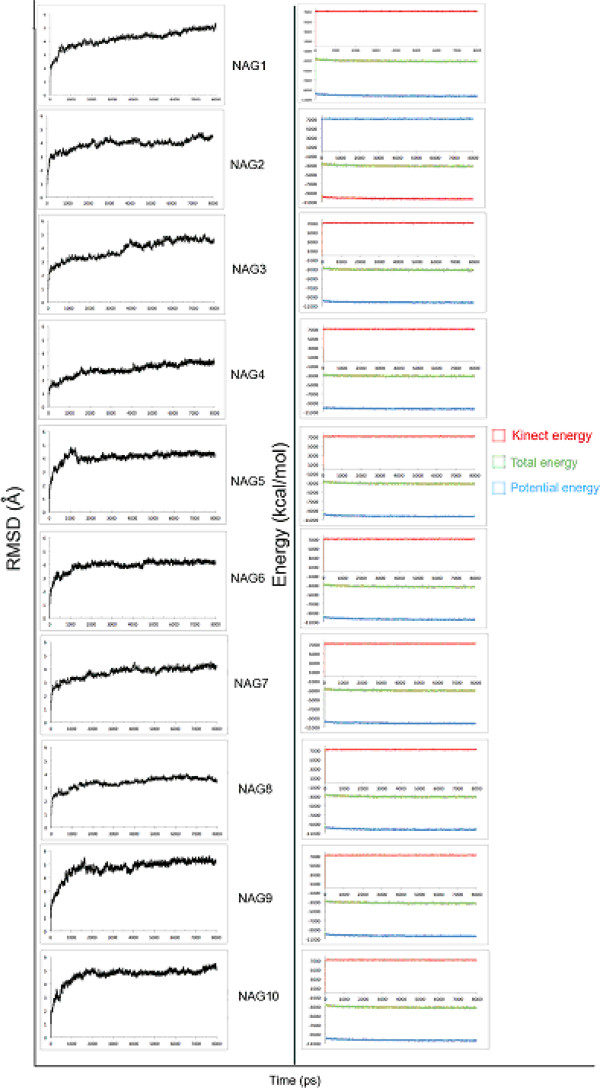
RMSD, total, energy and kinetic energies of then complexes after 8000ps of MD simulateion.

MD simulations were carried out for ten ligands. All were submitted to 8000 ps of MD simulations, as specified in the methodology. The MD determines the trajectories of representative points in the space through the numerical solution of the equations of motion [22]. The basic objective of this method is to observe the evolution of the system through these equations, because it is through the interactions among the particles that the system is able to maintain both mechanical and thermal equilibrium, and if there is any external perturbation, the system tends to reach a new equilibrium [22]. The results of MD are depicted in Figure 3, which shows the variation in the RMSD of Cα atoms for the protein during the simulation time. The RMSD was dependent on each complex, with the lowest variation observed for the complexes NAG4 (3.8 Å) and NAG8 (3.8 Å) and the greatest for NAG9 (5.8 Å) and NAG10 (5.8 Å); the larger molecules showed the largest fluctuations. All simulations of the complex reached equilibrium after 4.000 ps of simulation. In addition, the kinetic, potential and total energies were also obtained throughout the simulations (Figure 3). As can be seen, there were no significant fluctuations. The initial increase in the kinetic energy is related to the heating step of the system; subsequently, it is kept constant during the simulation. This indicates that the temperature of the system was constant at the value determined for the study. The potential energy, and hence the total energy, remained constant throughout the trajectory, indicating a relaxation of the system; therefore, equilibrium was reached for all compounds.

The average structure from MD simulation of each compound was used to determine the binding energy. In each MD simulation, a cut was made in the equilibrium region. The average structure was separated into COMP (UNAcP-NAG), LIG (NAGs) and PTN (UNAcP), and each was, finally, optimized again. Thus, the binding energy was determined by the formula ΔG = E_COMP_ - E_PTN_ - E_LIG_. As a result, NAG5 and NAG2 showed the best affinity for UNAcP (Table 1), differing by only 6.7 kcal/mol. In contrast, the compound NAG6 showed the lowest interaction with UNAcP (Table 1). The docking results differ considerably from MD simulations. In docking methodology, only ligand is flexible in a rigid active site. This neglects the conformational changes induced by ligand. Conversely, the MD simulations permit the molecular motion of target and ligand. Hence, our docking results were refined by MD simulation, improving the accuracy of the model. In other words, all docking experiments should follow MD or quantum mechanics and molecular mechanics (QM/MM) calculations.

**Figure 4:**
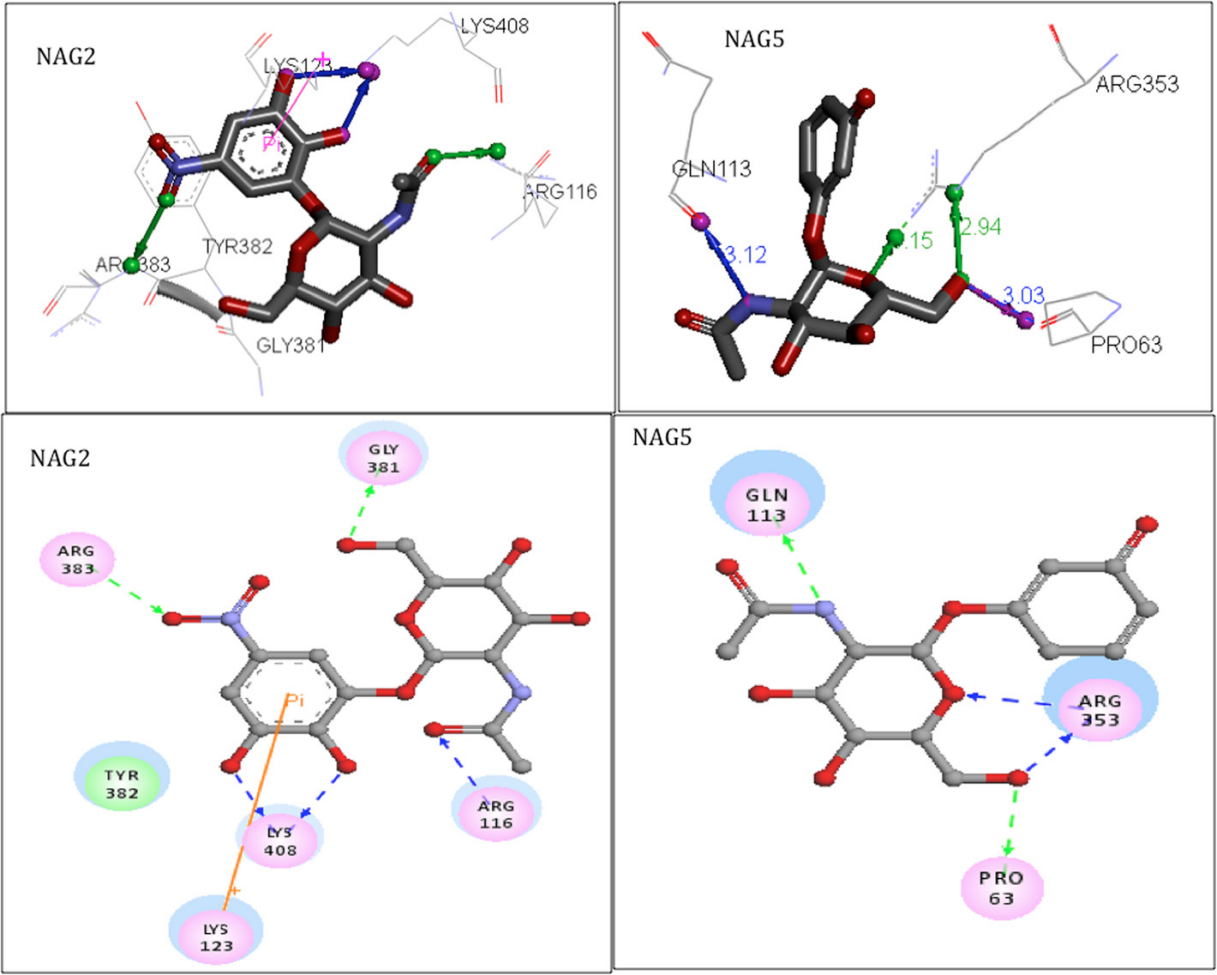
**Interactions formed between compounds with NAG5 and NAG6 pyrophosphorylase enzyme.** Green line: HBA, Blue Line: HBD, Rose Line: hydrophobic interactions and Yellow line: π-cation interaction. Visualization generated by the program Discovery Studio 3.5.

The most active compound designed (NAG5) formed with UNAcP hydrogen bonds (HB), π-cation and hydrophobic interactions. Figure 4 highlights in 2D and 3D the pharmacophoric conformation. As can be seen, the intermolecular interaction for NAG5, HB network was formed with Arg116, Gly381, Arg383 and Lys408 residues, with the distance ranging from 2.9 Å to and 3.14 Å. The hydrophobic interaction was observed with the aromatic ring of Tyr382 with a distance of 3.85 Å. Moreover, the aromatic ring of NAG5 also interacted with the Lys123 through a π-cation interaction, with a distance of 3.99 Å. However, NAG6, the least active compound designed, while showing intermolecular interactions very similar to those of NAG5, differs in the absence of hydrogen bonds formed between vicinal hydroxyl groups (catechol) with Lys123. This result suggests the hydroxyl is important as a pharmacophoric group, but not essential for intermolecular recognition among UNAcP and inhibitors. In addition, hydrogen bonds are formed between ligand and Pro63, Gln113 and Arg353, which ranged from 2.94 Å 3.12 Å with NAG6.

## Conclusions

The search for an effective control for witches’ broom has demanded much effort in recent years, mainly due to the socio-economic impact that the plague has had in the state of Bahia and other areas that were previously cocoa producers. This study was developed with a goal of finding a compound that can be used for effective control of witches’ broom. For this purpose, a structure-based drug design approach was used to study the molecular recognition between UNAcP of *M. perniciosa* and substrates. Initially, the molecular target chosen was pyrophosphorylase enzyme, and ten ligands were finally designed to inhibit the enzyme and thereby prevent the development of the witches’ broom fungus. Furthermore, the docking results were refined by MD simulations, improving the accuracy of the models.

The next step in developing new strategies for the control of witches’ broom is the *in vitro* enzyme pyrophosphorylase assay of these compounds, which can be synthesized by classical carbohydrate chemistry. This work is in progress and will be reported in due time.

## Methods

Initially, the previously built [14] UNAcP model was structurally aligned with the crystal structure of pyrophosphorylase with PDB code 1JV1 [16], whose active site was preserved, as shown by the structural alignment [23]. Next, the atomic coordinates of the substrate were transferred from the protein crystallographic 1JV1 to the UNAcP model. A redocking process was performed to verify the ability to reproduce the intermolecular interaction of the docking method. Next, a set of carbohydrate derivatives with *N*-acetyl moiety (Figure 1) were docked against the UNAcP model. The docking studies were conducted using AutoDock Vina 1.1.2. The search algorithm used was Iterated Local Search Global Optimizer for global optimization. In this process, a succession of steps with a mutation and local optimization (the method of Broyden-Fletcher-Goldfarb-Shanno [BFGS]) were conducted, and each step followed the Metropolis criterion [24]. To explore all active sites, the researchers constructed a grid box, which was defined as a cube with the geometric center between *N*-acetylglucosamine-1-phosphate and UTP, with dimensions of 18x14x16 Å, spaced points of 1 Å and X, Y and Z coordinates of −58.323, 79.633 and −18.926, respectively.

The complexes formed in the docking step were minimized by SANDER routine using the AMBER 10 software [25]. The complexes between ligand and molecular targets were optimized by 500 cycles for each algorithm, steepest descent (SD) and conjugate gradient (GG), with spatial restriction of the ligand atoms of 500 kcal/mol. This initial optimization aims to remove the interactions caused by molecular packing and improve the fit of the ligand in the protein, once the docking simulation considers the protein as a rigid structure. Finally, minimization of 3000 cycles (1500 SD and 1500 GG) was performed using the same specifications as above, but without spatial restriction.

Following the minimizing step, the complexes were submitted to molecular dynamics simulations (MD). The initial simulations were 100 picoseconds, varying the temperature until the system reached a final temperature of 300 K. After the heating step, the complexes were submitted to MD of 8000 picoseconds at a constant temperature of 300 K. All minimization and MD simulations were carried out using the Generalized Born implicit solvent model, with cut-off value of 14 Å. The results were analysed using Root-Mean-Square Deviation (RMSD) graph per simulation time.

The program ptraj was used to separate the remaining balance from the zone of the trajectory [26]. The use of the entire trajectory can generate less reliable results. The same program was used to calculate the average structure (MS). MS was subdivided into complex (_COMP_), protein (_PTN_) and ligand (_LIG_); all were submitted to 3000 cycles of optimization. The results of this step were used to determine the binding energy through the following equation: Binding energy = E_COMP_ - E_PTN_ - E_LIG_.

## Competing interests

We declare that there are no any competing interests.

## Authors’ contributions

All authors contributed equality for the development of the manuscript. MCSJ, SAA, AGN, AAD and AGT contributed with docking and molecular dynamics simulations, search for natural inhibitors, sequence of enzyme, high performance computation environment, design of inhibitors, analysis of results and elaboration of manuscript, respectively. All authors read and approved the final manuscript.
